# Dynamic Response of the cbbL Carbon Sequestration Microbial Community to Wetland Type in Qinghai Lake

**DOI:** 10.3390/biology12121503

**Published:** 2023-12-07

**Authors:** Ni Zhang, Kelong Chen, Siyu Wang, Desheng Qi, Zhiyun Zhou, Chuanyou Xie, Xunjie Liu

**Affiliations:** 1 Qinghai Province Key Laboratory of Physical Geography and Environmental Process, College of Geographical Science, Qinghai Normal University, Xining 810008, China; zhangni0224@163.com (N.Z.); 15229584811@163.com (S.W.); 2018116@qhnu.edu.cn (D.Q.); 13897423633@163.com (Z.Z.); 2 Key Laboratory of Tibetan Plateau Land Surface Processes and Ecological Conservation, Ministry of Education, Qinghai Normal University, Xining 810008, China; 3 National Positioning Observation and Research Station of Qinghai Lake Wetland Ecosystem in Qinghai, National Forestry and Grassland Administration, Haibei 812300, China; 4 Key Laboratory of Refrigeration Technology, Tianjin University of Commerce, Tianjin 300134, China; 17865567606@163.com (C.X.); 18437918911@163.com (X.L.)

**Keywords:** Qinghai–Tibet Plateau, climate change, carbon cycle, carbon sequestration microorganisms, carbon fixation

## Abstract

**Simple Summary:**

In this paper, the differences in carbon sequestration microbial communities in different wetland types and their main influencing factors are investigated. It was found that Proteobacteria was the dominant group of carbon sequestration microorganisms in the marsh wetland and river source wetland, and Cyanobacteria was the dominant group of carbon sequestration microorganisms in the lakeside wetland. The α diversity, relative abundance of Proteobacteria and total carbon content of the marsh wetland were the highest, followed by the river source wetland and lakeside wetland. The soil temperature and moisture and the total carbon content were the most important factors affecting the structures of carbon-sequestering microbial communities. Compared with the riverhead and lakeside wetlands, the marsh wetland has the highest carbon storage.

**Abstract:**

The soil carbon storage in the Qinghai–Tibet Plateau wetlands is affected by microbiota and wetland types, but the response mechanisms of carbon sequestration microorganisms on the Qinghai–Tibet Plateau to different wetland types are still poorly described. To explore the differences in carbon sequestration microbial communities in different wetlands and the main influencing factors, this study took a marsh wetland, river source wetland and lakeside wetland of Qinghai Lake as the research objects and used high-throughput sequencing to study the functional gene, cbbL, of carbon sequestration microorganisms. The results showed that the dominant bacterial group of carbon sequestration microorganisms in marsh and river source wetlands was Proteobacteria, and the dominant bacterial group in the lakeside wetland was Cyanobacteria. The alpha diversity, relative abundance of Proteobacteria and total carbon content were the highest in the marsh wetland, followed by the river source wetland, and they were the lowest in the lakeside wetland. In addition, the physical and chemical characteristics of the three wetland types were significantly different, and the soil temperature and moisture and total carbon content were the most important factors affecting the community structures of carbon-sequestering microorganisms. There was little difference in the total nitrogen contents between the marsh wetland and river source wetland. However, the total nitrogen content was also an important factor affecting the diversity of the carbon sequestration microbial community. In summary, the wetland type significantly affects the process of soil carbon sequestration. Compared with the riverhead and lakeside wetlands, the marsh wetland has the highest carbon storage.

## 1. Introduction

Wetlands play an important role in the regulation of the global carbon balance in terrestrial ecosystems [1]. A third of the soil carbon pool (450–530 Pg) is reportedly stored in wetlands that cover only 5–8% of the global land surface [2,3]. Therefore, maintaining high carbon storage in wetland ecosystems is important to mitigate global warming caused by the increase in atmospheric carbon dioxide concentrations [4]. Previous studies have found that the main influencing factors of wetland soil carbon storage include climate factors [5,6], litter quantity and quality [7] and soil physicochemical properties [8,9]. However, the soil carbon storage in wetlands is also heavily controlled by the wetland type [10]. As the “engine” of the biogeochemical cycle, microorganisms drive the carbon cycle of wetland soil through catabolism and anabolism [11]. In addition, studies have found that the CO_2_ sequestration potential of microorganisms depends on the type of ecosystem [12,13], which further confirmed the close relationship between microbial communities and wetland types. As an important part of carbon sequestration in wetlands, autotrophic microorganisms take up 8–27% of the organic carbon pool in wetlands [13,14]. The Calvin–Benson–Bassham cycle is the main mechanism of CO_2_ uptake by photoautotrophs and chemoautotrophs [15,16], and its first step is catalyzed by ribulose 1,5-diphosphate carboxylase/oxygenase (RubisCO) [17]. This enzyme regulates the Calvin cycle rate, and its large subunit is encoded by the cbbL gene [18]. Therefore, it is necessary to explore the community change characteristics of cbbL functional flora in different types of wetlands.

With an average elevation of more than 4000 m, the Tibetan Plateau is a driving force and amplifier of global climate change [19]. It has the largest area of alpine wetlands in the world [20]. Located in the northeast of the Qinghai–Tibet Plateau, the Qinghai Lake Basin is an important barrier for maintaining the ecological security of the local basin [21] and a national nature reserve with internationally important wetlands [22]. However, the response mechanisms of carbon sequestration microorganisms in different wetland types in the Qinghai Lake Basin are still unknown. In this study, the river source wetland, lakeside wetland and marsh wetland in the Qinghai Lake Basin were selected as the research objects, the cbbL functional gene microbial flora was determined by high-throughput sequencing technology and the biogeochemical properties of the soil were also determined. Our aim was to (1) study the response patterns of carbon sequestration microbial communities to different wetland types, (2) assess the effects of soil properties driven by different wetland types on soil microbial communities involved in carbon sequestration and (3) analyze the interaction between carbon sequestration microorganisms and environmental factors in different wetland types in the Qinghai Lake Basin. The results can not only provide basic data for the quantitative study of the carbon cycle and transformation in the Qinghai Lake Basin but also provide a reference and guidance for the study of the mechanisms governing carbon sources and sinks in alpine wetlands.

## 2. Materials and Methods

### 2.1. Overview of the Study Area

The Wayan Mountain (37°43′–37°46′ N, 100°01′–100°05′ E) is a typical river source wetland with an average elevation of 3720–3850 m, an average annual temperature of −3.31 °C and an average annual precipitation of 420.37 mm. The vegetation is dominated by *Kobresia humilis*. Xiaobo Lake (36°41′–36°42′ N, 100°46′–100°47′ E) is flooded year-round and is a typical swamp wetland with an average elevation of 3228 m, an average annual temperature of −0.8–1.1 °C and an average annual precipitation of 324.5–412.8 mm. The dominant species are *Kobresia tibetica* and *Blysmus sinocompressus*. Bird Island (36°57′–37°04′ N, 99°44′–99°54′ E) is a typical lakeside wetland with an elevation of 3194–3226 m, an average annual temperature of −0.7 °C and an average annual precipitation of 322.7 mm. *Allium przewalskianum*, *Astragalus adsurgens* Pall and *Poa annua* L. are the dominant species in this wetland type.

### 2.2. Soil Sample Collection

Soil samples were collected in June 2020 (early growing season). For each plot (1 m × 1 m), 0–10 cm of topsoil (4.5 cm diameter of soil drill) was collected using a five-point sampling method. According to the name of the experimental station, the samples were named Wck (Wayan Mountain), Bck (Xiaobo Lake) and Nck (Bird Island). Five replicates were collected at each sample point, and a total of 15 soil samples were collected. All samples were mixed and sieved (pore size was 2 mm), part of the soil samples was stored in liquid nitrogen tanks for soil DNA extraction and the remaining samples were stored on ice packs for immediate return to the laboratory.

### 2.3. Determination of Soil Physical and Chemical Properties

The TDR-300 (Spectrum Technologies, Plainfield, IL, USA) was used to monitor soil (0–10 cm) moisture, and the LI-8100 instrument (LI-COR, Lincoln, NE, USA) was used to monitor soil (0–10 cm) temperature. Soil pH was measured with a pH meter (FE20-FiveEasy pH, Mettler Toledo, Gießen, Germany) after the soil/water ratio was mixed at 1:2.5. The air-dried soil was screened with 100 mesh and then weighed and wrapped with 20 mg tin foil, and the total carbon (TC) and total nitrogen (TN) were determined by an Elemental Analysis System (Vario EL III, Elemental Analysis System GmbH, Langenselbold, Germany).

### 2.4. DNA Extraction and Illumina MiSeq Sequencing

Soil microbial DNA was extracted from 0.5 g of fresh soil using a PowerSoil DNA Isolation Kit (Mio-bio, Carlsbad, CA, USA). Using cbbL-specific primers, the forward primer (5′-GACTTCACCAAAGACGACGA-3′) and reverse primer (5′-TCGAACTTGATTTCTTTCCA-3′) were used to increase carbon sequestration by organisms [23]. The PCR products were sequenced using the Illumina MiSeq sequencing platform. DNA extraction and quantification and PCR procedures were performed as previously described [23].

### 2.5. Statistical Analysis

The MicrobiotaProcess_1.6.2 package was used, where the ggrarecurve function plotted the dilution curve, the get_alphaindex function calculated the microbial diversity index (ACE index, Chao1 index, Simpson index, Shannon index) and the get_pca function calculated Beta diversity. The Wilcoxon rank sum test was used to calculate the significance between the groups. The operational taxonomic unit (OTU) annotation result was used to organize the plot through R 4.1.2. Functional groups of microorganisms were predicted by FAPROTAX [24]. The aov function of the Stats package performed variance analysis on the data. The mantel_test function of the linkET_0.0.2.10 package in R4.1.2 calculated the data correlation and *p*-values, and the qcorrplot function drew the correlation network heat map. The UpsetR_1.4.0 package’s upset function drew the OTU histogram, the pheatmap package drew the correlation heatmap, and the rest of the pictures were drawn by ggplot2_3.3.5.

## 3. Results

### 3.1. Changes in the Environmental Variables of Different Wetland Types

**Figure 1:**
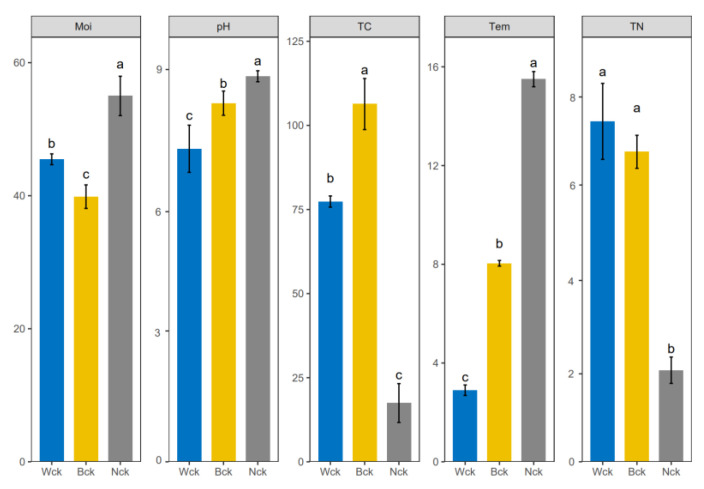
Variations in soil physicochemical factors in different wetland types; abc indicates significance, the same letter indicates no significant difference between groups (*p* > 0.05) and different letters indicate a significant difference between groups (*p* < 0.05). Tem: soil temperature, Moi: soil moisture, TN: total nitrogen, TC: total carbon, pH: soil pH.

The soil’s physical and chemical factors were affected by wetland types, and there were significant differences in the spatial distances (Figure 1). The temperature, moisture and pH of the lakeside wetland were significantly higher than those of the marsh wetland and river source wetland. The soil moisture of the river source wetland was higher than that of the marsh wetland, but the soil temperature and pH were lower than those of the marsh wetland. The total carbon and nitrogen contents of the Qinghai Lake Wetland were the lowest, the total carbon content of the marsh wetland was higher than that of the river source wetland and the total nitrogen content of the river source wetland was higher than that of the marsh wetland.

### 3.2. cbbL Sequencing and Microbial Community Diversity Characteristics of Different Wetland Types

**Figure 2:**
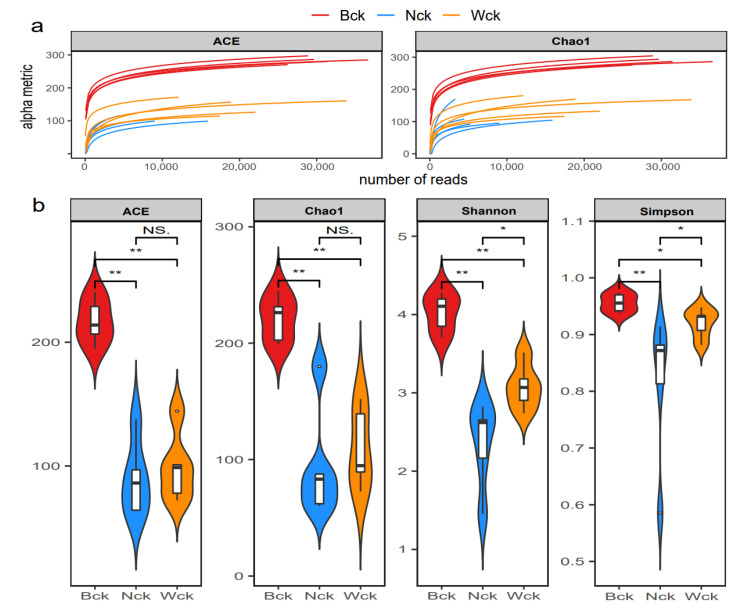
The diversity of carbon sequestration microorganisms in Qinghai Lake; (**a**) sample dilution curve; (**b**) alpha diversity index. NS indicates *p* > 0.05, * indicates *p* < 0.05 and ** indicates *p* < 0.01.

**Figure 3:**
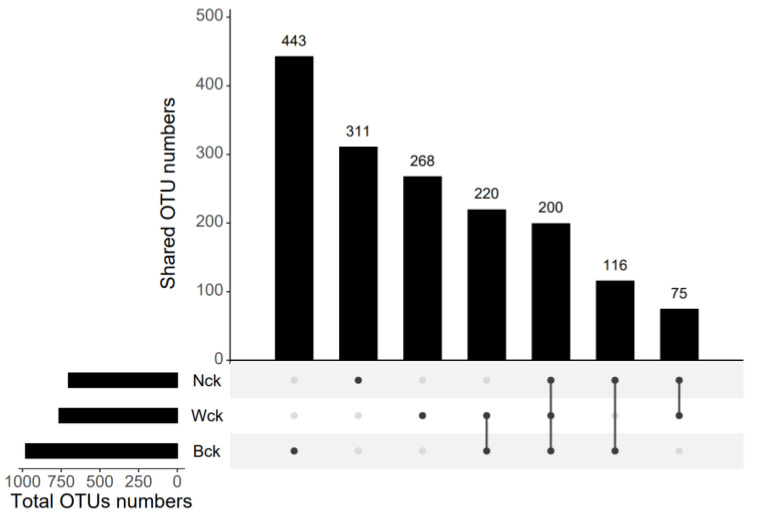
OTU distribution of each group.

**Figure 4:**
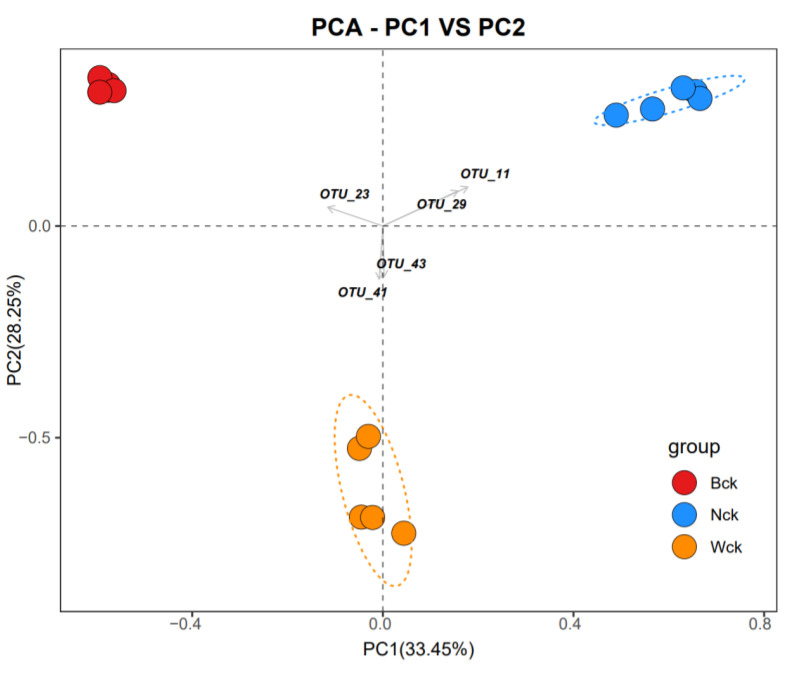
Principal component analysis (PCA).

The sequencing results showed that the amount of sequencing data was reasonable and that the depth was sufficient to cover the species diversity of the samples (Figure 2a). The total OTUs of the cbbL microflora in the marsh wetland, lakeside wetland and riverhead wetland were 979, 702 and 763, respectively; the numbers of unique OTUs were 443, 311 and 268, respectively; and the number of common OTUs among the three wetland types was 200 (Figure 3). Changes in the wetland type significantly altered the alpha diversity index of the cbbL community (Figure 2b). The species richness of the marsh wetland was much higher than that of the lakeside and riverhead wetlands, and the difference in species richness between the lakeside wetland and riverhead wetland was small. However, the Simpson index and Shannon index of the three wetland types were significantly different, with the largest value for the marsh wetland and the smallest value for the lakeside wetland. The PCA results (Figure 4) showed that there were significant differences in the aggregation tendency among the groups, and soil heterogeneity was small in the marsh wetland.

### 3.3. Changes in Soil cbbL Community Structure in Different Wetland Types

**Figure 5:**
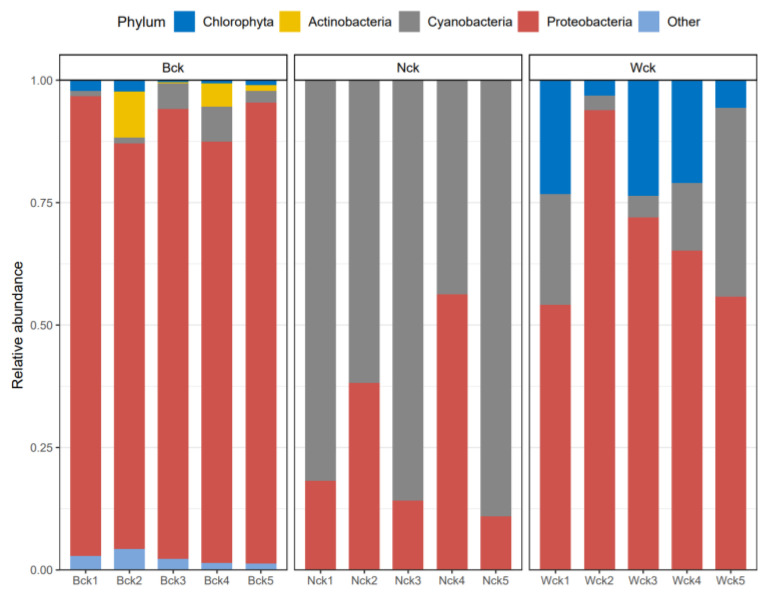
Level structure of cbbL carbon sequestration microbial phyla.

**Figure 6:**
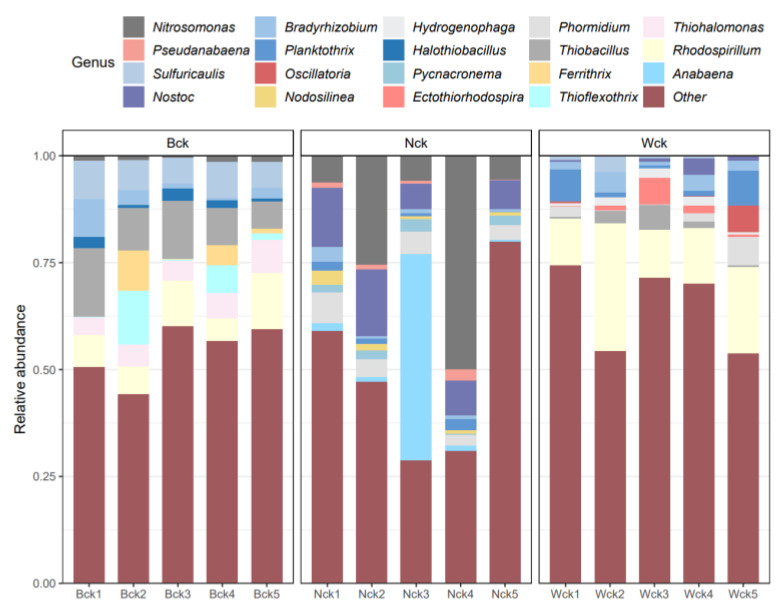
Structure and composition of cbbL carbon sequestration microorganisms at the genus level.

Proteobacteria was the main bacterial group in the carbon sequestration microflora in the marsh and river source wetlands (Figure 5). Compared with the marsh wetland, the relative abundance of Proteobacteria in the river source wetland decreased by 21.89%. However, in the soil samples of the lakeside wetland, Cyanobacteria (71.71%) was the main bacterial group (Figure 5), and the relative abundance of Proteobacteria in this wetland type only accounted for 27.34%. In addition, the ANOVA showed that the relative abundances of Proteobacteria and Cyanobacteria in the lakeside wetland were significantly different from those in the other two wetlands (*p* < 0.05). As shown in the column chart of the genus-level flora (Figure 6), the relative abundances of unclassified florae in the marsh, lakeside and riverhead wetlands were 53.46%, 48.49% and 63.84%, respectively. There were nine species of dominant bacteria (relative abundance > 1%) in the marsh wetland and lakeside wetland, and the relative abundance of *Thiobacillus* (10.73%) in the former was the highest, while that of *Nitrosomonas* (18.37%) was the highest in the latter. In the Heyuan wetland, there were 10 dominant bacterial groups at the genus level (relative abundance > 1%), and *Rhodospirillum* (16.78%) was the dominant bacterial group with the highest relative abundance. The three bacterial groups showed significant differences in the different wetland types (*p* < 0.05).

**Figure 7:**
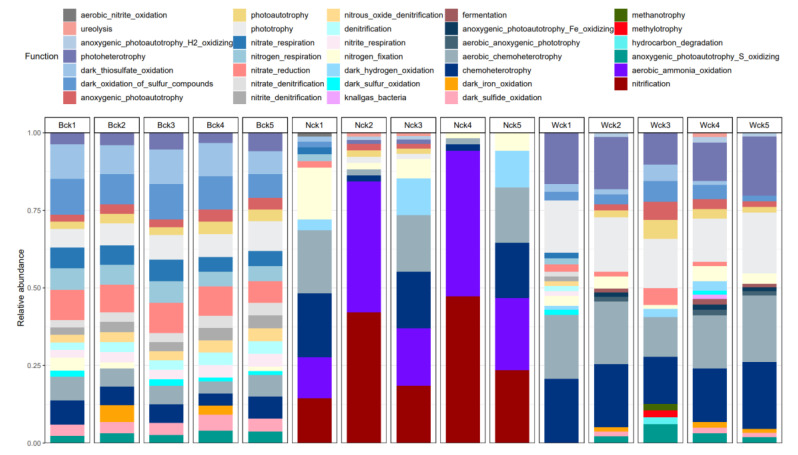
Carbon sequestration microbial functional groups in the Qinghai Lake Wetland.

**Figure 8:**
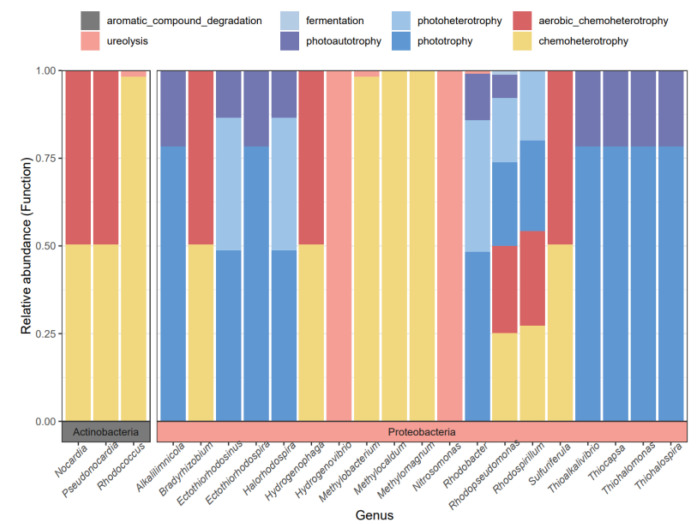
Carbon cycle process mainly performed by the horizontal flora.

The results of the functional annotation of cbbL functional flora in the Qinghai Lake Wetland showed that the main ecological functions of carbon sequestration microorganisms in the three types of wetlands (relative abundance > 0.1%) were divided into 34 functional groups (Figure 7). In addition to carbon sequestration by the Calvin cycle, many bacteria in the soil carbon sequestration bacterial community in the Qinghai Lake Wetland are also closely related to the oxidation of nitrogen and sulfur compounds. The carbon sequestration microbial functional groups in the marsh were mainly dark_oxidation_of_bitter _compounds, dark_thiosulfate_oxidation, nitrate_reduction, phototrophy and chemoheterotrophy, aerobic_chemoheterotrophy, nitrogen_respiration, nitrate_respiration, photoheterotrophy and dark_sulfide_oxidation. The microbial functions of carbon sequestration in the lakeside wetlands were mainly nitrogen_respiration and nitrate_respiration. The carbon sequestration microbial functions in the Heyuan wetland mainly focused on chemoheterotrophy, aerobic chemoheterotrophy, phototrophy and photoheterotrophy. There are eight functional groups related to the carbon cycle (Figure 8). The reverse positioning of the microbial flora related to the carbon cycle shows that 22 genus-level florae belonging to two phyla are mainly involved in the biochemical cycle of carbon elements. Chemoheterotrophy or phototrophy are the main functions of most of the florae, and some of the florae participate in the carbon cycle mainly through ureolysis (Figure 8).

### 3.4. cbbL Microbial Diversity in Different Types of Wetlands

**Figure 9:**
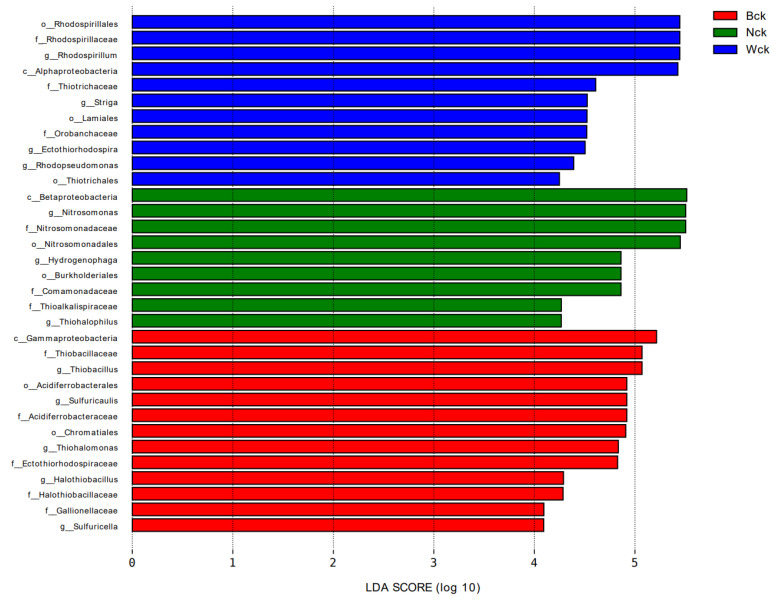
Different classification levels of bacteria in three wetland types.

To identify the differential bacterial florae of carbon-sequestering microorganisms at different levels in the three wetland types, we performed a differential discriminant analysis using LEfSe (LDA > 4). Thirty-three taxa, including three classes, seven orders, 11 families, and 12 genera, showed statistically significant differences (Figure 9). Specifically, *Thiobacillus*, *Sulfuricaulis*, *Thiohalomonas* and *Halothiobacillus* occupy dominant positions horizontally in marsh wetlands. *Nitrosomonas* showed absolute advantages in the lakeside wetland, while *Rhodospirillum*, *Ectothiorhodospira* and *Hydrogenophaga* showed obvious advantages in the riverhead wetland. These microflorae can often be used as biomarkers to indicate grouping.

### 3.5. Correlation between cbbL Community Characteristics and Environmental Data in the Qinghai Lake Wetland

**Figure 10:**
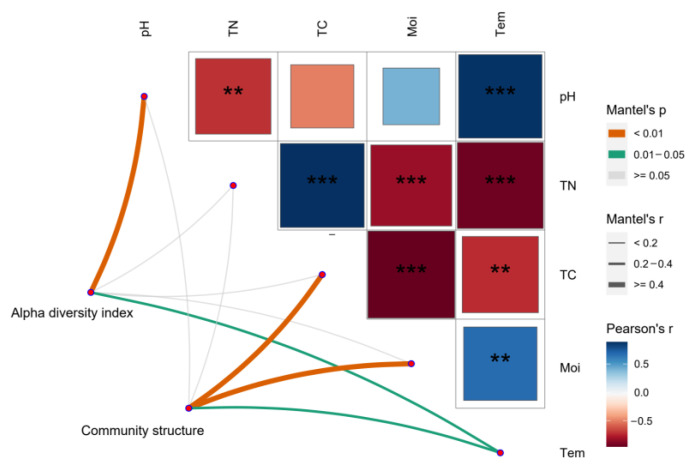
Heatmap of the correlation between carbon sequestration microbial community characteristics and soil physicochemical factors. ** indicates *p* < 0.01 and *** indicates *p* < 0.001; Tem: soil temperature, Moi: soil moisture, TN: total nitrogen, TC: total carbon, pH: soil pH.

**Figure 11:**
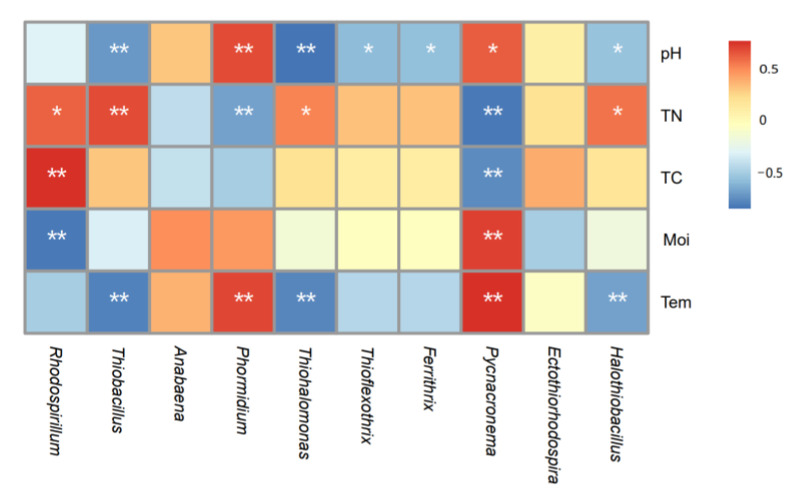
Correlation analysis of horizontal carbon sequestration microorganisms and soil physicochemical factor. * indicates *p* < 0.05, and ** indicates *p* < 0.01; Tem: soil temperature, Moi: soil moisture, TN: total nitrogen, TC: total carbon, pH: soil pH.

The interaction of soil physicochemical factors (Table S1) in the Qinghai Lake Wetland jointly affected the characteristics of the soil carbon sequestration microbial community (Figure 10). Specifically, the alpha diversity index of carbon-sequestering microorganisms was significantly affected by the soil pH, total nitrogen and temperature, and the dominant microflora of carbon-sequestering microorganisms was significantly affected by the soil temperature, moisture and total carbon content (Figure 10). A further analysis of the relationship between the dominant flora at the genus level and environmental factors (Figure 11) showed that *Pycnacronema* was extremely sensitive to the soil microenvironment, and its relative abundance was significantly affected by various factors, while *Ectothiorhodospira* and *Anabaena* were not significantly responsive to changes in the microenvironment. In addition, the relative abundance of *Rhodospirillum* was closely related to the total carbon, total nitrogen and soil moisture (Figure 11), and its sensitivity to the soil pH change was relatively low, while *Ferrithrix* and *Thioflexothrix* were mainly affected by the pH. The relative abundance of the other flora was significantly affected by the soil pH, total nitrogen and soil temperature. The total carbon content in the soil was significantly negatively correlated with *Pycnacronema* and positively correlated with *Rhodospirillum*. This result shows that the carbon storage of the Qinghai Lake Wetland is closely related to the relative abundance ratio of these two types of bacteria.

## 4. Discussion

### 4.1. Effects of Wetland Type on cbbL Carbon Sequestration Microbial Community Diversity

Microbial diversity is an important factor affecting the structure and functional stability of biological communities [25] and is often used as an indirect indicator of carbon sequestration potential [26]. This study found that the alpha diversity of the carbon sequestration microbial community in Qinghai Lake was significantly affected by the heterogeneity of wetland types and their hydrological effects, which was largely attributed to the differences in the soil environment shaped by wetland types [10,27]. Fang et al. studied the differences in microbial community structures in the sediments of lakes, rivers and wetlands in the Dongping Lake Basin and confirmed the influence of the wetland type on the microbial alpha diversity [28]. In addition, the species richness index and evenness index of the marsh wetland are much higher than those of the lakeside wetland and riverhead wetland, but there is little difference in the species richness between the lakeside and riverhead wetlands, which may be due to the high total carbon content in wetlands, promoting the reproduction of carbon sequestrating microorganisms [29,30]. Previous studies found that due to long-term overhumidity and anoxic environments, the organic matter in marshes could not be completely decomposed and accumulated in large quantities [31]. The total carbon contents of the lakeside wetland and river source wetland are significantly lower than that of the marsh wetland, which further supports this view. This study also found that the diversity of the carbon-sequestering microbial community was not only significantly affected by the soil pH and temperature but also closely related to the total nitrogen content. Liao et al. studied the diversity of carbon sequestering microbial communities in salt marsh wetlands and found that it was mainly positively correlated with the soil’s organic carbon and total nitrogen contents, and negatively correlated with the soil particle size [32]. Wang et al. studied the community characteristics of autotrophic CO_2_-fixing bacteria in karst wetland groundwaters with different nitrogen levels, and it was found that the dissolved inorganic nitrogen was significantly negatively correlated with the diversity index of the cbbL gene-containing bacterial community [33]. However, Wang et al. studied the abundance and diversity of carbon sequestration bacteria communities in the soil ecosystem of a karst wetland and showed that there was no statistically significant relationships between the community diversity of carbon sequestration microorganisms and soil environmental factors [34]; this result was different from that of carbon sequestration microorganisms in the Qinghai–Tibet Plateau. Wang studied the factors affecting the diversity of the carbon sequestration microbial community under changes in precipitation in the Qinghai–Tibet Plateau and argued that the soil temperature, moisture and pH were the most important influencing factors, which differs somewhat from the results in this study [35]. However, the study by Wang on the influencing factors of carbon sequestration microbial communities in the Qinghai–Tibet Plateau showed that changes in the total nitrogen content significantly affected carbon sequestration microorganisms, providing support for the results of this study [36].

### 4.2. Effects of Wetland Type on cbbL Carbon Sequestration Microbial Community Structure

Changes in the community abundance of carbon sequestration microorganisms are also often used to indirectly characterize the carbon sequestration potential [37]. Previous studies have confirmed that there are differences in the composition of carbon sequestration microbial communities in wetland ecosystems [26,34]. This study found that the wetland type significantly affected the carbon sequestration microbial community structure in Qinghai Lake. Fang et al. reached the same conclusion by studying the differences in the microbial community structures in the sediments of lakes, rivers and wetlands in the Dongping Lake Basin [28]. Ma et al. studied the carbon sequestration microbial communities of four wetland types in the Hulun Lake Basin and found that there were significant differences in the relevant microbial communities of different wetland types [38]. Proteobacteria is a vital microbe in the global carbon cycle, which may have an important impact on soil nutrient cycling and even biogeochemical processes [39,40]. Proteobacteria is the main bacterial group in the carbon sequestration microflora from the marsh and river source wetlands of Qinghai Lake. Liao et al. studied the response of the soil carbon sequestration bacteria community to the changes in the plant community in a coastal marsh wetland and found that Proteobacteria was the most important carbon sequestration bacteria group [32]. Ma et al. also found that Proteobacteria was the most important carbon sequestration bacteria in different wetland types [38]. The above conclusions are consistent with those of this study. However, the relative abundance of Proteobacteria in the lakeside wetland was low, and the most dominant bacterial group was Cyanobacteria. It may be that the limited light in marsh wetlands and riverhead wetlands affects the growth and reproduction of photoautotrophic microorganisms such as Cyanobacteria [41].

There were significant differences in the genus-level dominant bacteria groups among the three wetland types. *Thiobacillus* was the dominant bacterium in the marsh wetland, *Nitrosomonas* was the dominant bacterium in the lakeside wetland, and *Rhodospirillum* was the dominant bacterium in the riverhead wetland. Previous studies also found that different wetland types had different dominant flora at the genus level. Wang et al. studied karst wetlands and found that *Bradyrhizobium*, *Hydrogenophaga*, *Mesorhizobium*, *Methylibium* and *Sulfuricaulis* were the dominant bacterial genera in the carbon-fixing bacterial community containing the cbbL gene [34]. Zhu et al. studied carbon sequestrating microorganisms in rice wetlands and found that at the genus level, *Cupriavidus*, *Thiobacillus* and *Thioalkalivibrio* were the top three in abundance [42]. It may be that the vegetation and hydrological effects of different wetlands create the unique wetland environment and cause the differences in the dominant flora of carbon sequestration microorganisms [28]. The soil temperature is the main driving factor of soil carbon sequestration microbial community structures [34,43], and microorganisms are also very sensitive to changes in the soil water availability [39]. In this study, it was found that the community structure of carbon sequesters in the three wetland types in Qinghai Lake was jointly regulated by the soil temperature and moisture and the total carbon content. The study by Ma et al. showed that the bacterial community structure and abundance related to CO_2_ fixation were changed mainly by changing the sediment carbon content in wetland types [38]. Wang et al. found that the main soil physicochemical factors affecting the community structure of soil carbon sequestration bacteria in karst wetlands were carbon components and the soil temperature [34]. The above results were consistent with this study. The relative abundance of Proteobacteria was consistent with the change in the total carbon content in the wetlands. It may be that the high nutrient environment of marsh wetlands provides an adequate habitat for carbon sequestration microorganisms [44].

## 5. Conclusions

Based on the high-throughput sequencing results of cbbL functional genes, this study revealed the dynamic response mechanism of carbon sequestration microbial community characteristics to three wetland types in Qinghai Lake. The alpha diversity index of carbon sequestration microbes was the highest in the marsh wetland, followed by the river source wetland and lakeside wetland. The wetland type significantly changed the dominant flora of the carbon sequestration microorganisms. The dominant flora of the carbon sequestration microorganisms in the marsh and river source wetlands was Proteobacteria, and Cyanobacteria was the main flora in the lakeside wetlands. In addition, the environmental factors of the three wetland types were significantly different, and the change trend in the total carbon content was consistent with the diversity index and the relative abundance of Proteobacteria. The type of wetland in the Qinghai Lake Basin might be regulated by vegetation and hydrological effects, which might change the environmental factors, and may further affect the distribution characteristics of carbon sequestration microbial communities. This study provides a new understanding of the carbon sequestration mechanism of alpine wetland ecosystems.

## Data Availability

The raw data have been uploaded to NCBI, and its BioProject is PRJNA1006286.

## Supplementary Materials

The following supporting information can be downloaded at: https://www.mdpi.com/article/10.3390/biology12121503/s1, Table S1: Soil physicochemical factor.
